# Developing Bilingualism in Nursing Students: Learning Foreign Languages beyond the Nursing Curriculum

**DOI:** 10.3390/healthcare9030326

**Published:** 2021-03-14

**Authors:** Luis M. Dos Santos

**Affiliations:** Woosong Language Institute, Woosong University, Daejeon 34514, Korea; luisdossantos@woosong.org; Tel.: +82-010-3066-7818

**Keywords:** bilingualism, health education, nursing curriculum, nursing education

## Abstract

Nursing curriculum usually focuses on vocational development to train students to become nursing professionals after graduation. However, due to the packed major schedule and curriculum, many students are not required to take additional foreign language courses for their associate degree. Based on the lens of social cognitive career theory, the researcher sought to understand the motivations and reasons behind the learning behaviours. One research question was guided in this study, which was, what are the motivations and reasons for taking foreign language courses beyond their (i.e., nursing students) major curriculum and coursework plan? A qualitative research method was employed to collect interview data from 60 nursing students. The finding of this study indicated that the interest in career development and personal consideration were two of the most important factors for foreign language learning for these groups of nursing students. The results of this study provided recommendations for college leaders, government agencies, and policymakers to reform and polish foreign language courses and offer directions to contemporary students of the nursing curriculum. Students may also be benefitted as the study outlined the motivations and reasons for foreign language learning. Therefore, all parties may take this study as a blueprint to exercise their future developments.

## 1. Introduction

### 1.1. Research Background 

Nursing students are trained to become professionals in the medical field. Communication to patients in their native language or targeted language may increase patients’ understanding [1]. Effective communication may also increase confidence in the treatment. However, learning a foreign language and culture is an interesting but difficult experience for some nursing students due to their packed and busy schedules. Besides governmental requirements, motivation and interest are among the reasons individuals decide to learn a foreign language [2,3]. 

In the United States, Spanish language courses are popular as Spanish is one of the most commonly used languages after English. For nearly 70 years, the United States Department of Education has contributed resources for foreign language development and education. Due to geographic and historical factors, many foreign-born people enter the United States for work and academic study [4]. Therefore, gaining proficiency in a foreign language may benefit university graduates for personal and professional development. As a result, according to a report from the Modern Language Association (MLA) of America, Spanish (790,756) was the most popular foreign language among university students in the United States in 2013, followed by French (197,757), American Sign Language (109,577), German (86,700), Italian (71,285), Japanese (66,740), Chinese (61,055), Arabic (32,286), Latin (27,192), and Russian (21,962) [5]. Based on a recent report [6], as Spanish and English share many linguistic and cultural similarities, American students are interested in pursuing Spanish language learning. Although English is a Germanic language and Spanish belongs to the Romance languages, they (i.e., English and Spanish) have similar pronunciation and lexical sets as both come from the European continent [7]. English speakers may find Spanish an easier foreign language to handle than Asian languages, such as Chinese, Japanese, Hindi, and Arabic [8]. Although Asian languages may be harder for many American students, this does not limit interest in learning due to various teaching and learning behaviours, desires, and approaches [9,10]. 

Knowledge of a foreign language and cultural awareness are two of the most important elements for nursing professionals to work with patients from different parts of the world [11]. Although some medical sites have introduced an electronic translator for immediate interpretation, patients tend to use face-to-face interpretation to avoid misunderstanding [12]. A recent report [13] indicated that students and professionals are not prepared for globalisation due to a lack of foreign language and cultural awareness. Eisenberg [14] indicated that foreign language courses are greatly needed in the contemporary American classroom, regardless of the school level. School leaders, policymakers, and government officials should also encourage students to learn at least one foreign language and cultural direction to improve their professional competitiveness [15]. Some scholars [16] have indicated that if secondary schools and universities do not set up the curriculum requirements for foreign language courses, American students tend not to take any foreign language courses. Although American families use nearly 400 different languages at home [17], foreign language learning is still not familiar to many United States residents. In contrast, most European students and professionals speak or have some proficiency in at least one foreign language [18]. Based on previous studies [19], American professionals are at a disadvantage as most are not bilingual or multicultural. Therefore, government officials and school leaders always try to offer different foreign language and cultural courses beyond the fixed curriculum for non-foreign-language major students [20]. 

There are different motivations and reasons why people decide to learn a foreign language, in this case, nursing students. First of all, some researchers [21,22,23] believe that interest in a foreign culture may increase the motivation to learn. One study [24] investigated how popular culture and the entertainment industry may influence foreign language learning. Using the attitude/motivation test battery theory, the researcher collected data from 120 students learning Korean as a foreign language in Thailand. The results indicated that the Korean Wave in the entertainment industry had strongly influenced how individuals made foreign language learning decisions [25]. More than 90 per cent of these Korean learners were female students who had wanted to learn the language due to their appreciation of Korean TV dramas and singers. Another study [26] also indicated that students, adults, and individuals tend to learn a foreign language due to popular culture and entertainment, such as the K-pop culture [25,27]. A recent study [28] asserted that many language learners want to study the Japanese language due to anime and manga. 

Some researchers [20,22,23,29] have linked the motivations and reasons for career development and opportunities. A previous study [11] investigated the motivations of a group of learners selecting Chinese as their foreign language within the One Belt One Road countries. The results indicated that most learners believed that the Chinese language would facilitate career development and bring business opportunities, as Chinese visitor numbers were increasing rapidly in their regions. Through the lens of social cognitive career theory, most participants stated that financial considerations are the most important motivation for foreign language learning [11]. Another recent study [28] indicated that foreign language learning always involves financial and economic considerations. For example, individuals who can speak a foreign language can work with people from international locations, such as visitors and tourists. The local government may also offer special financial and career opportunities for residents who master a foreign language [20]. According to Gardner [30], individuals tend to learn a foreign language due to their personal interests and career opportunities. First, unlike foreign language major students, who have extensive interest in particular foreign languages and cultures, students in other subject majors, such as nursing and engineering, may take foreign language courses due to academic requirements [31]. Some individuals have also stated that foreign cultural understanding allowed them access to business opportunities in other parts of the world [32]. In addition, others plan to live in a foreign country for either study or work after university graduation. Preparation for a foreign culture could be an advantage [33]. In short, different individuals have their own reasons and motivations. 

### 1.2. Theoretical Framework: Social Cognitive Career Theory 

Social cognitive career theory was employed to investigate the motivations and reasons for taking foreign language courses beyond the major curriculum and coursework plans of community college students majoring in nursing. Social cognitive career theory is a useful theory to understand the problems and social phenomenon, including students’ university major selection [34], doctoral candidates’ learning behaviours [35], the mental and psychological well-being of professionals [36], first-generation college students’ behaviours [37], adolescent’s understanding about their personal choice and selection [38], and second career-changing professional [39]. As the nature of social cognitive career theory is useful for interdisciplinary studies [36,40,41], the researcher may employ this theory to the problem of foreign language learning among community college students in the United States [39]. 

**Figure 1:**
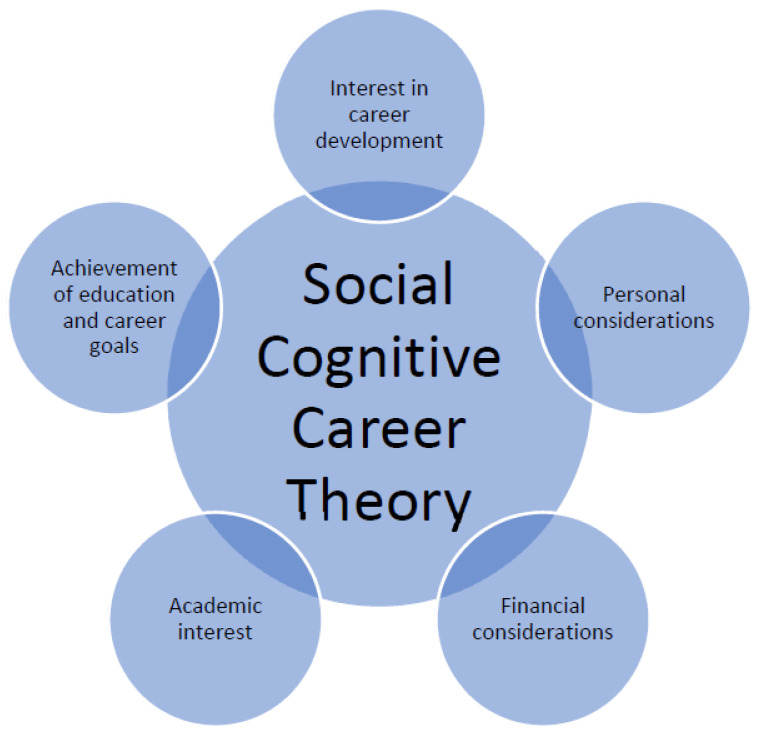
Social cognitive career theory.

Social cognitive career theory was developed from Albert Bandura’s social cognitive theory [42]. The theory advocates that self-efficacy and social situation influence the behaviours of people. Based on the social cognitive theory concept, social cognitive career theory focuses on the direction and intention of the personal factors and decisions of self-efficacy, outcome expectations, and personal achievements as these elements relate to career development [40]. Social cognitive career theory was originally developed with three major factors, including (a) interest in career development, (b) achievement of education and career goals, and (c) academic interest. However, due to the development of social communities and the changing human behaviours, different researchers and scholars have edited and revised the theory with different perspectives over the decades [40,43,44,45]. Currently, the researcher employed social cognitive career theory with five different factors of influences, including (a) interest in career development, (b) achievement of education and career goals, (c) academic interest, (d) financial considerations, and (e) personal considerations [35,43,44,46,47,48,49]. For details, please refer to Figure 1. 

### 1.3. Purpose of the Study 

As bilingualism and multi-language skills become important for medical professionals, this study examined the role of motivations and reasons for nursing students’ learning in the United States. The researcher concluded five points about why this study would benefit medical professionals, nursing educators, and school leadership. First of all, readers of this study will be beneficial because this study outlines the current curriculum problems in a nursing study programme. In fact, many of the current nursing programmes do not have a strong focus on developing bilingual skills and abilities. Therefore, this study’s results became the encouragement and motivation to polish the current nursing study curriculum. 

Second, this study’s results collected feedback and voices from a group of nursing students who want a reform in their nursing study programme. Although college and university leaders and administrators always accept their students’ voices, the feedback is usually disorganised. The results of this study merged and analysed the feedback as a professional study. School leadership may use this study as a blueprint to polish their current curriculum plan(s). 

Third, as nursing education is one of the vocational-based programmes that focus on practical skills and hands-on experience, school leadership tends to upgrade student-internship and vocational skill-oriented problems. However, it is important to upgrade communication and language skills, as nursing professionals need to interact with their patients in the clinical environment. Therefore, this study’s results become significant for further encouragement of general education training (e.g., for nursing students). 

Fourth, adding foreign language courses to the nursing curriculum would be difficult due to the nursing study programme’s packed schedule. Based on nursing students’ voices in this study, the school administrators should understand how to balance the foreign language elements and vocational-based skills into the nursing study programme. 

Fifth, selecting the types of foreign language courses is essential. In other words, should the college select and add the general foreign language course or the foreign language course for medical and nursing students (e.g., foreign language with a specific purpose)? The readers of this study will benefit as the nursing students provided comprehensive feedback for directive development. 

Learning a foreign language is not easy for all students, as many do not major in a foreign language programme. Therefore, it is important to understand the motivations and reasons behind their decision and decision-making process. This study was guided by one research question:

(1) Based on the lens of social cognitive career theory, what are the motivations and reasons for taking foreign language courses beyond a learner’s major curriculum and coursework plan (i.e., community college students majoring in nursing)?

## 2. Materials and Methods

This study examined full-time traditional undergraduate students in nursing who were enrolled in a community college in the United States’ Midwest region. Most secondary school curricula require that high school graduates take at least two semesters of foreign language study to graduate. However, it is not uncommon that these graduates cannot speak these learned foreign languages after several learning terms. On the other hand, students may select another foreign language at the university level. In short, individuals choose to learn a foreign language based on various elements and factors. 

A qualitative research methodology [50] was employed for this study. The study aimed to collect understanding and feedback from the participants about their motivations and reasons for foreign language learning at a community college in the United States. As the study sought to collect the data and materials from a single site with a group of individuals with a similar background, the researcher decided to use the case study research design [51] as a direction. In this study, the researcher focused on the experience and feedback of a group of nursing students (i.e., with foreign language learning background and intention) at a community college (i.e., single site) in the United States. One of the advantages of the case study methodology is the narrowed focus. Unlike other qualitative research methodologies, such as phenomenological analysis with a larger focus on the community and region situation [52], the case study researcher tends to focus on the problem and situation in a targeted site and group. Therefore, the study’s finding may focus on the site’s particular outcomes or some situations and sites with a similar background and problem.

### 2.1. Participants and Recruitment

First of all, the populations of participants may be a concern for some qualitative research studies. According to Creswell [53], an effective qualitative research study may involve with at least 50 participants. In this study, 60 participants were invited to this study. Therefore, the current study met the recommendation (i.e., for the effective population). The researcher contacted community college administrators and department heads for the potential study based on previous connections. Both agreed with the arrangement. Therefore, the researcher sent the invitation letter (i.e., email about the rationale of the study), protocol (i.e., interview protocol), and related material to the community college staff. Secondly, the community college staff sent the invitation to nursing college students who are currently taking foreign language courses. Based on the administrator’s feedback, the invitation was sent to 60 students, and all agreed to participate. Thirdly, the participants contacted using the email on the invitation letter for further action. The researcher sent the content form and related materials to the participants for the data collection procedure. Finally, all these participants were traditional-aged freshmen and sophomore-year (i.e., first-year and second-year) students at the community college level. No non-traditional, returning, evening, and adult students were included. 

### 2.2. Data Collection

Due to the COVID-19 pandemic and the recommendation of social distancing from both community college leadership and government agencies, the researcher could only conduct online interview sessions for the data collection procedure. First of all, the researcher sent the email invitation letter, interview questions, and information about the overall procedure and the potential time to be taken for each participant’s interview. The participants responded to the email for confirmation. Secondly, the researcher sent out the link to the participants’ email address for the online interview session. The researcher used the Zoom application as the means to conduct the online interview sessions. During the interview sessions, the researcher employed a digital recorder for recording. Each participant agreed with this arrangement. However, no facial expressions and visual materials were marked. Each interview session lasted 62 to 87 min. For the interview question, please refer to Appendix A. Thirdly, member-checking interviews were conducted after the researcher completed the data analysis. Each participant agreed with their sharing and approved the study. Each member-checking interview lasted 23 to 42 min. 

### 2.3. Data Analysis 

After completing the 60 online interview sessions, the researcher transcribed the oral materials. The researcher re-read the written transcripts multiple times to collect and categorise the themes and groups. First of all, the researcher employed the open-coding technique to categorise the massive sharing into groups and directions based on the lens of social cognitive career theory and the research question. At this stage, the researcher grouped 12 themes and 13 subthemes. 

Secondly, based on the recommendations of different qualitative researchers, further analysis was required. Therefore, the researcher employed the axial-coding technique to narrow down the above-mentioned themes and subthemes. As a result, the researcher eventually grouped two themes and three subthemes as a finding. For details, please refer to Table 1. 

### 2.4. Human Subject Protection 

Personal privacy and data protection are the most important factors in this study. Therefore, the researcher employed all factors to protect all parties’ personal information and materials, including the participants, the community college, the administrators, the department heads, and the staff. Therefore, all the participants took a pseudonym for the study. The gender, in-depth background, visa status, and cultural heritage were not collected in order to protect the personal background and exercise the rationale of the study. As the community college, location, and background of the community college personnel did not influence the finding and results of the study, the researcher did not report (i.e., masked) the information of these parties. 

All the signed consent forms, personal contact, oral sharing, written transcripts, email links, and computer and related materials were locked in a passport-protected cabinet. Only the researcher had the rights to read the information. After the study was completed, the researcher deleted all the related materials to protect all parties’ personal privacy. The current study was supported by the Woosong University Academic Research (2019/2020/2021/1-12). 

## 3. Results and Discussion 

Based on the lens of social cognitive career theory [35,43,44,46,47,48,49], the researcher conducted interview sessions with nursing community college students in the United States, focusing on their motivations and reasons for studying a foreign language, and then categorised two themes and three subthemes for this study. For details, please refer to Table 1. 

### 3.1. Career Development 

Based on social cognitive career theory [35,43,44,46,47,48,49], individuals and groups may select a direction and language based on their career development potential. The idea of career development and opportunities was a motivation for foreign language learning. All participants expressed different levels of interests (i.e., foreign language learning) due to their career development and job opportunities after community college and university in the future. 

In other words, all connected their interest in foreign language learning to career development as a whole factor for motivation [54,55]. According to a previous study [56], large numbers of traditional-aged, non-traditional, returning, evening, and adult (NTREA) [57] students take foreign language courses to improve their personal competitiveness. Based on social cognitive career theory [13,14,15,16,17,18,19], the researcher may confirm that interest in career development highly influenced the participants’ motivation and reasons for foreign language learning. Particularly, all participants agreed that the ability to speak at least one foreign language might provide them with additional advantages in their job-seeking efforts after graduation. The researcher captured several comments:

*… many bilingual and English as a Second Language individuals are competing with us for the job opportunities … although English is the common language in the United States, people should speak additional languages … to upgrade our skills …*

[P#45]

*… I think foreign language will open up my opportunities in the future … if I can speak Spanish or German, I can go to Europe for master degree education in nursing … the market in other countries and regions are actually bigger than the United States … I want to open my eyes and see the international opportunities …*

[P#3]

*… if I don’t speak any foreign languages … many employers may not read my resume as there are many stronger candidates in the market … if I can speak Spanish, I can serve additional patients and community members, both in person and over the phone … at least I stand out in front of the crowd …*

[P#35]

#### 3.1.1. Opportunities for Major Subject

Many participants indicated that their academic major and department encouraged them to take additional foreign language courses beyond their set curriculum with an eye to career developments in the future. For example, a group of sophomore-year nursing students indicated that their department counsellors strongly encouraged them to take at least two to three years of foreign language courses to serve patients with an international background. As reflected by some previous studies [31,58,59,60], all healthcare professionals may encounter international and foreign patients in their workplace. These studies indicated that hospitals and medical facilities need to serve many international patients and patients who do not speak English as their native language. If medical professionals can speak a foreign patient’s language, misunderstandings and medical mistakes may be avoided without needing an interpreter to be present [61,62]. Some significant sharing was captured:

*… there are many international patients in our city … as a nursing student … I want to speak at least one foreign language to serve my patients from different cultural backgrounds … there are some keys words or basic conversations that medical professionals should know … so the patients will feel comfortable after the operations …*

[P#22, Nursing]

*… I learned Chinese at high school and Japanese at community college now because we have a strong Asian population … understanding and language and culture of the patients always increase the effectiveness of the treatment …*

[P#16, Nursing]

Almost all nursing students expressed a similar idea about how a foreign language may upgrade their professional services, language skills, and overall performance for non-English speakers in their career development and future workplace after community college and university. Based on the direction of social cognitive career theory, the motivations and reasons may relate to the interest in career development and the idea of helping international patients. Particularly, language and communication between patients and medical staff are key factors for treatments. With a strong background in the patients’ language and culture, they may receive upgraded services and treatments [31,58,59].

Two studies [63,64] have indicated that many students think that foreign language learning might open additional doors and opportunities in career promotion, particularly in public health promotion for new immigrants. Based on the social cognitive career theory [13,14,15,16,17,18,19], the researcher may confirm that the following sharing has a strong connection with the framework. A large group of nursing students with a concentration in health promotion shared:

*… health promotion always involves foreign patients … if I can speak additional languages, I can transfer my public health knowledge to all American, immigrants, international students, and foreigners …*

[P#1]

*… if I want to do nursing and public health promotion in Mexico or Mexican groups in the southern states … I better speak Spanish … although English may work in some cases, I think Spanish language will open up my opportunities and doors in South America …*

[P#11, International Business]

*… New York, London, and Hong Kong are three of the biggest cities globally … I wish I can go to Hong Kong for job and development … I learn Chinese and Chinese culture at college … so I can be well-prepared for this opportunity after university …*

[P#13, International Business]

In line with social cognitive career theory [35,43,44,46,47,48,49], almost all participants asserted that career development was always their motivation for learning a foreign language during their community college voyage. Most of the participants in health promotion programmes advocated that foreign language skills and proficiency always offer them opportunities to learn foreign culture at the school level—in other words, a well-prepared cultural background before joining the profession. As shown in some previous studies [30,65], students consider foreign language and culture proficiency as additional skills to improve and upgrade their academic programme. Many of these participants indicated that understanding a foreign language and culture may increase their job satisfaction and effectiveness due to the positive communication between different parties.

#### 3.1.2. Opportunities for Study Abroad 

Due to globalisation and freedom of movement, many students decide to study at international universities at both undergraduate and graduate levels [66,67,68]. However, according to some previous studies [8,69], besides some English-speaking countries and universities, most programmes are foreign-language-oriented. Therefore, students may not enrol or apply for either exchange or study-abroad opportunities without at least intermediate-level language proficiency [70]. Based on social cognitive career theory [35,43,44,46,47,48,49], the researcher may confirm that the motivation and reasons for foreign language learning may connect to the interest in study abroad in this study. Within this subtheme, many participants expressed that foreign language proficiency may increase their chance of academic opportunities to study abroad. Several pieces of feedback were captured:

*… I want to go to Brazil for a year-long study abroad opportunity … but I need at least two years of Portuguese proficiency to apply for the programme … so I study very hard on top of my packed schedule … but the Brazilian opportunity and Portuguese knowledge must increase my career development in the future …*

[P#7]

*… I love the East Asian culture … I learn Chinese because I plan to apply for the exchange programme to Taiwan … I want to go to Taiwan before I transfer my associate degree to my third year of study at the state university … but without any Chinese background, the programme wouldn’t accept my application …*

[P#8]

In conclusion, according to a previous study [71], many students tend to develop themselves as multicultural learners to apply to a study abroad programme and experience the culture and background of the host country. In line with social cognitive career theory [35,43,44,46,47,48,49], career development, study abroad, and the connection between an academic major in nursing and foreign language were important among these community college students. The findings also show that the desire to upgrade their skills and personal development is linked to the motivations and reasons for learning a foreign language beyond their packed schedules. 

### 3.2. Personal Interests 

The United States is one of the countries that involves individuals and groups from different parts of the globe. As noted above, a previous report [17] indicated that nearly 400 spoken languages are currently used in the United States. Regardless of geographic location, proficiency in a language other than English is important for many American students. Although many countries and organisations have a great number of individuals and groups who can speak English, without a strong background in the local language and culture, students may not handle local living standards [72]. With the lens of social cognitive career theory [35,43,44,46,47,48,49], the researcher may confirm that the following sharing matched the framework’s direction of personal interest. The researcher captured two interesting points shared on ideas about the living experience:

*… I want to learn the culture and people’s experience in the hosted country … not just about university experience … If I can go to Japan, I want to make some friends in Japan, but if I don’t understand what should be experienced in Japan, I cannot understand what should be explored …*

[P#19]

*… I learn a foreign language due to my personal interests … I think interest … is the key for learning … I learn nursing because I like it. I study foreign language because I like it … Without the motivation and desire … I will not continue with my education …*

[P#56]

From the perspective of social cognitive career theory, based on the participants’ thoughts, it can be said that a large proportion believed that learning a foreign language should not be limited to the classroom environment but also incorporate an understanding of the local community with their personal interests. With the reflection of a previous study [28], a foreign language always involves cultural knowledge and background experience of speaking the language. As for the participants’ feedback, they were gaining a basic background in the foreign language and its culture, which may increase their likelihood of joining the host communities and countries in the future [73]. 

#### Family Heritage

As noted by a previous study [74], the United States is a country with a diverse population due to people’s immigration from many countries. In other words, many students or their parents were born in a foreign country. Therefore, many of these students desire to understand the language and culture of their heritage. Based on the lens of social cognitive career theory, the researcher may confirm that their motivation and reasons of foreign language learning may connect to their ideas about family heritage. Community college offers opportunities for these students to gain such insight. The researcher captured several stories:

*… the college offers the Spanish language for in nursing direction and general direction … as a Peruvian American, I want to gain my heritage … this is one of the reasons why I want to study Spanish beyond my major in nursing … In fact, learning Spanish does not only link to or with my job or internship in the hospital … more importantly, I think Spanish can connect my personal background, family tree, and my roots in Peru … I have two backgrounds, one as an American, but more importantly, I am a Peruvian …*

[P#21, Nursing]

*… I think culture and my personal background always influence my motivation of Spanish learning … my family came from Mexico 17 years ago … I can speak Spanish but not Spanish with Specific Purposes, such as business Spanish … I want to learn…because of my background and history …*

[P#55, Communication]

*… my family, my friends, my community, my cousin … are from Haiti … but my family tried their very best … not speak Spanish with me as they think I should learn English … therefore, I only have lower level of Spanish skills … not at the professional level … therefore, I want to take some Spanish courses at the upper level … I want to learn the Central and South American culture and background … as I am from these areas …*

[P#56, Communication]

According to social cognitive career theory [35,43,44,46,47,48,49], personal interests always influence individuals’ choices and decision-making processes. With the reflection of some previous studies [22,75,76], individuals and groups are influenced by different factors in second language learning, whether or not it is linked to heritage. Most participants believed that personal development directed their motivations and reasons for learning beyond their academic major [77]. More importantly, the desire to live overseas and connect with their own heritage directed their foreign language choice(s) [8]. 

## 4. Conclusions, Implications, and Future Research Directions 

Foreign language learning is one of the most important communication and language skills for many contemporary learners, regardless of age, background, educational opportunity, location, and desire. Nursing college students learn an additional foreign language due to a desire for career development and personal considerations. As the United States is one of the biggest countries in the world and home to many diverse ethnic groups and communities (i.e., individuals who speak different languages at home), community colleges, government agencies, and policymakers should continue to upgrade the current regulation for foreign language development and education to meet the desires and needs of the population. More importantly, residents who can speak additional foreign languages will improve the country’s competitiveness [65]. Therefore, encouraging students and potential future leaders to learn foreign languages must be a priority in the current educational environment. 

### 4.1. Implications 

The findings of this study will be beneficial in two areas. First of all, community college leaders and departmental heads may understand that they should reform and promote their foreign language programmes and courses to meet the desires and demands of their students, particularly for nursing students. Many students indicated their interest in international travel, career development, and study abroad. Foreign language departments may design courses with specific purposes for these targeted groups of students.

Second, government agencies and policymakers may reform the budgets and policies relevant to community college students and graduates who transfer to a university after graduation, particularly the nursing programme. For example, the government may further develop current regulation and policy to meet the needs of these different groups of students. 

### 4.2. Limitations and Future Research Directions 

First of all, this was a case study focusing on a single community college in the United States. However, as the United States is one of the world’s biggest countries, students from different regions may have very different ideas and understandings. Future research studies may expand the horizon of this research to different regions and community colleges. The broader and more holistic picture generated may include additional voices from different parties with various perspectives. 

Secondly, this study employed online interview sessions due to the COVID-19 pandemic and social distancing recommendation. Therefore, face-to-face interactions, field note marking, and item exchanging were not possible. In the future, researchers and scholars should upgrade this study with these approaches to gather additional data collection and materials to enrich the overall density of the findings. 

## Figures and Tables

**Table 1:** Themes and subthemes of the study.

Themes and Subthemes
Career Developments
Opportunities for Major Subject
Opportunities for Study Abroad
Personal Interests
Family Heritage

## Data Availability

Not applicable.

## Appendix A

The Interview Questions

1. What foreign language are you studying?
2. Why do you want to study this foreign language? Can you tell me more?
3. Did you study this foreign language before community college? If so, when and why? If no, why this foreign language now?
4. What make(s) you want to study this foreign language now? Can you tell me more?
5. Why do you want to study this foreign language during community college? Not high school? Not university? Not in the future? Why now?
6. Will this foreign language help you in the future in your opinion? How and why?
7. Do you think this foreign language is useful in your community? How and why?
8. If you can speak this foreign language fluently, how would it help you in the future?
9. Can you use this foreign language in your daily practice? How and why?
10. Do you think this foreign language can help you with your career development and job opportunities? How and why?
11. Follow-up questions.
